# Automatic detection, classification, and quantification of sciaenid fish calls in an estuarine soundscape in the Southeast United States

**DOI:** 10.1371/journal.pone.0209914

**Published:** 2019-01-16

**Authors:** Agnieszka Monczak, Yiming Ji, Jamileh Soueidan, Eric W. Montie

**Affiliations:** 1 Department of Natural Sciences, University of South Carolina Beaufort, Bluffton, South Carolina, United States of America; 2 Department of Mathematics and Computational Science, University of South Carolina Beaufort, Bluffton, South Carolina, United States of America; University of Auckland, NEW ZEALAND

## Abstract

In the Southeast USA, major contributors to estuarine soundscapes are the courtship calls produced by fish species belonging to the family Sciaenidae. Long-term monitoring of sciaenid courtship sounds may be valuable in understanding reproductive phenology, but this approach produces massive acoustic datasets. With this in mind, we designed a feature-based, signal detector for sciaenid fish calls and tested the efficacy of this detector against manually reviewed data. Acoustic recorders were deployed to collect sound samples for 2 min every 20 min at four stations in the May River estuary, South Carolina, USA from February to November, 2014. Manual analysis of acoustic files revealed that four fish species, belonging to the family Sciaenidae, were the major sound producers in this estuarine soundscape, and included black drum (*Pogonias cromis*), silver perch (*Bairdiella chrysoura*), spotted seatrout (*Cynoscion nebulosus*), and red drum (*Sciaenops ocellatus*). Recorded calls served as an acoustic library of signature features that were used to create a signal detector to automatically detect, classify, and quantify the number of calls in each acoustic file. Correlation between manual and automatic detection was significant and precision varied from 61% to 100%. Automatic detection provided quantitative data on calling rates for this long-term data set. Positive temperature anomalies increased calling rates of black drum, silver perch, and spotted seatrout, while negative anomalies increased calling rates of red drum. Acoustic monitoring combined with automatic detection could be an additional or alternative method for monitoring sciaenid spawning and changes in phenology associated with climate change.

## Introduction

Soundscape ecology is a rather new research field in marine ecology that strives to understand the temporal rhythms and spatial patterns of sound in marine environments. In estuaries, soundscapes are rich in biological (biophony) and non-biological origins (geophony and anthrophony) [1]. In the Southeast United States, significant contributors to estuarine soundscapes are snapping shrimp (genus *Alpheus* and *Synalpheus*) and fish that belong to the family Sciaenidae, which include black drum (*Pogonias cromis*), silver perch (*Bairdiella chrysoura*), spotted seatrout (*Cynoscion nebulosus*), red drum (*Sciaenops ocellatus*), Atlantic croaker (*Micropogonias undulatus*), and weakfish (*Cynoscion regalis*) [2–11]. These fish calls are species-specific due to morphological differences in swim bladder and sonic muscle combinations and are used primarily for courtship purposes [3, 6, 12–15]. Efficient analysis of long-term acoustic data sets can assist in understanding and detecting changes in fish reproductive behavior, which may help with assessing the health of estuaries. Thus, there remains a need to develop signal detectors that identify and quantify fish calls in order to shorten the time of analysis when collecting large acoustic data sets. However, the diversity of fish acoustic signals amidst a background of biological (e.g. snapping shrimp snaps, other fish calls, and dolphin vocalizations), anthropogenic (e.g. boat noise), and physical (e.g. rain and waves) sounds presents a significant challenge for signal detectors to effectively classify and quantify fish calls by species.

Advances in automatic signal detection have enabled the detection of underwater bioacoustic signals originating from crustaceans [9, 16], amphibians [17], and marine mammals [18–23]. However, there are few studies where signal detectors are used to detect fish acoustic signals, and even fewer studies that use a signal detector to identify fish calling in acoustically rich soundscapes. Recently, the application of automatic call recognition assisted in identifying striped cusk-eel (*Ophidion marginatum)* calls in Boston Harbor, MA, USA; Lusitanian toadfish (*Halobatrachus didactylus)* vocalizations in the Tagus estuary, Montijo, Portugal; red grouper (*Epinephelus morio*) calls on the West Florida Shelf, FL, USA; and boatwhistle calls of oyster toadfish (*Opsanus tau*) in Harris Creek Oyster Sanctuary in Chesapeake Bay, MD, USA [24–29]. Currently, there are no automatic signal detection approaches for sciaenids, which dominate estuaries in the Southeast USA and are important ecologically and economically to the recreational fishing industry.

In this study, we developed a signal detector that identifies and quantifies calls of four fish species that belong to the family Sciaenidae. The specific objectives were to (i) create an acoustic library of calls and characteristic call features for black drum, silver perch, spotted seatrout, and red drum; (ii) using these data, design an automated signal detector for these four fish species and apply this signal detector to acoustic files recorded for a nine month time span at four recording stations located in the May River estuary, South Carolina, USA; (iii) compare results from the signal detector to the results obtained from manual analysis; and (iv) investigate spatial, temporal, and environmental factors that influence species-specific calling rates for both automatic and manual detection datasets. These data are providing a foundation for automated signal processing that will assist with long-term analysis of reproductive behaviors for a community of fish living and contributing to an estuarine soundscape. We hope this detector is applicable in other estuarine soundscapes, and we encourage other researchers to contact us to test this detector.

## Materials and methods

**Fig 1:**
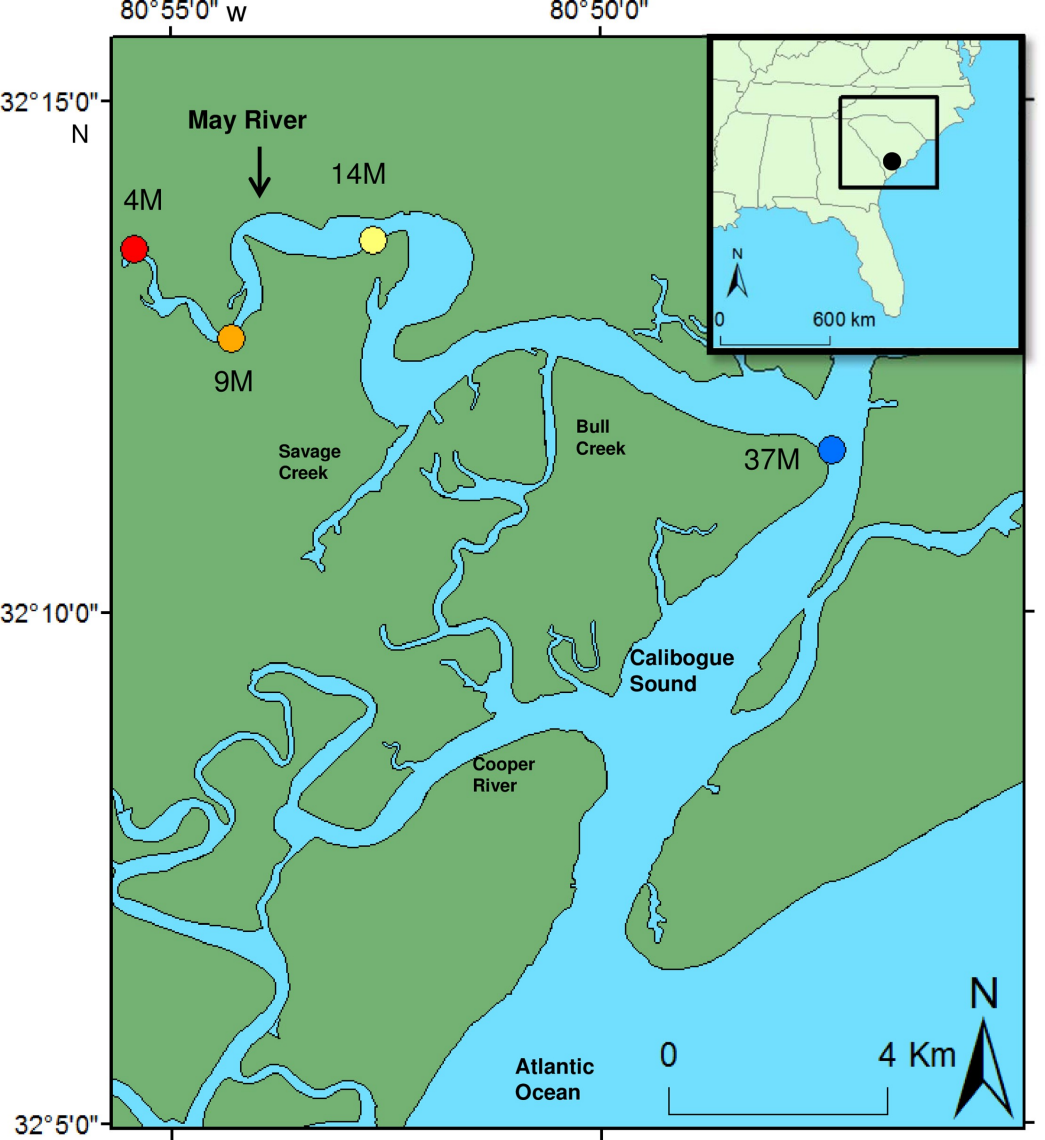
Location map. Locations of stations 4M, 9M, 14M, and 37M that were acoustically monitored from February 26 to November 21, 2014 in the May River estuary, South Carolina, USA. Station 4M (red circle) was located near the source and station 37M (blue circle) was located at the mouth of the tidal river. (Inset) Map of the May River, South Carolina (black circle) showing the approximate location of this large tidal river in reference to the east coast of the United States.

We conducted nine months of acoustic monitoring of the May River (32°12’49”N; 80°52’23”W), South Carolina, a large subtidal river estuary that is approximately 22.10 km long and 0.01 km wide near the source and 1.00 km wide at the mouth (Fig 1). The water depth increases from the source (~3 to 7 m) to the mouth (~4 to 18 m) and changes with semi-diurnal tides (~2.5 to 3.1 m). Along the river, there are smaller subtidal creeks (i.e. Savage Creek, Bull Creek, and Bass Creek) and numerous intertidal creeks. Bordering the river and creeks are intermittent oyster rubble and live oyster reefs composed of the eastern oyster (*Crassostrea virginica)* and broad areas of salt marsh composed of smooth cord grass (*Spartina alterniflora)*.

### Acoustic data collection

We deployed acoustic recorders (DSG-Oceans, Loggerhead Instruments, Sarasota, FL, USA) to monitor the estuarine soundscape at four locations (i.e. 4M, 9M, 14M, and 37M) based upon previous work (Fig 1) [8, 11]. We deployed an acoustic recorder at station 4M because this location exhibited minimal calling activity of fish. We deployed acoustic recorders at stations 9M, 14M, and 37M because data revealed that those locations contained large chorusing aggregations of silver perch, spotted seatrout, and / or red drum. Permission for deployment of recorders was granted by the South Carolina Department of Natural Resources. Field studies did not involve endangered or protected species.

We mounted DSG-Ocean recorders in custom built instrument frames (Mooring Systems, Inc., Cataumet, MA, USA) with attached water level and temperature loggers (HOBO 100-Foot Depth Water Level Data Logger U20-001-02-Ti and HOBO Water Temperature Pro v2 U22-001, Onset Computer Corporation, Bourne, MA, USA) that were placed in PVC housing. Temperature loggers were scheduled to record water temperature every 1 h, while depth loggers were scheduled to record water level every 10 min. The instrument frames, DSG-Oceans, and PVC logger housings were spray painted with antifouling paint (Trilux 33, West Marine, Hilton Head Island, SC, USA). The instruments, mounted in the frames, were then deployed on the bottom of the river ~10 m from the shoreline. This deployment method was accomplished by attaching a 7 m galvanized chain to the instrument frame. The chain was then attached to a line, which stretched along the river bottom to the side of the marsh following methods described previously [11].

The DSG-Ocean recorder was equipped with a High Tech Inc. hydrophone (i.e. sensitivity of -186 dBV μPa^-1^) with a flat frequency response between ~0.1 and 30 kHz. The system is calibrated by the manufacturer with a 0.1 V (peak) frequency sweep from 2–100 kHz, and it is powered by 24 D-cell alkaline batteries housed in a PVC tube (i.e. 0.7 cm length, 11.5 cm diameter). In this study, the DSG-Oceans were scheduled to record the soundscape for 2 minutes every 20 minutes at a sampling rate of 80 kHz. Acoustic recordings were saved as DSG files on a 128 GB SD card. DSG files were then downloaded and batch converted into wav files using the DSG2wav software (Loggerhead Instruments, Sarasota, FL, USA). HOBO logger data were downloaded using HOBOwarePro software (Onset Computer Corporation, Bourne, MA, USA). DSG-Oceans were then outfitted with new batteries and reassembled in instrument frames with HOBO loggers for redeployment following methods previously described [11].

### Manual detection

During this study, we collected a total of 69 696 wav files over three deployments in the nine month time frame Table 1. Each 2 min wav file was manually reviewed in Adobe Audition CS5.5 software (Adobe Systems Incorporated, San Jose, CA, USA). Spectrograms were visually analyzed using a spectral resolution of 2048 (i.e. the number of vertical bands used to draw frequencies in the Adobe Audition spectrogram). Calls of four sciaenid fish species (i.e. black drum, silver perch, spotted seatrout, and red drum) were manually detected during the analysis. The calls were identified by comparing acoustic recordings to spectrograms published in previous studies [2, 4, 8, 11, 14, 15, 30]. An observer scored each 2 min wav file based upon the intensity of calling for each fish species. The calling intensity score was based on four categories (i.e. 0 = no calls; 1 = one call; 2 = multiple calls; 3 = overlapping calls or chorus) following previous methods [3, 11]. During manual review, we did not count the number of calls in each acoustic file because of the challenge associated with overlapping calls when fish were chorusing.

**Table 1:** Summary of deployment and retrieval information of acoustic DSG-ocean recorders in the May river estuary, South Carolina, USA.

Station	Deployment No.	Start date (mm:dd:yy)	End date (mm:dd:yy)	Days sampled	No. of files collected
4M, 9M, 14M, 37M	1	02/26/14	05/22/14	86	24768
4M, 9M, 14M, 37M	2	06/05/14	08/15/14	72	20736
4M, 9M, 14M, 37M	3	08/30/14	11/21/14	84	24192
** **	TOTAL	02/26/14	11/21/14	242	69696

### Automatic detection

Based on the acoustic recorder used in this study, the following method was used to calculate received sound pressure level (SPL):

$$S=h+g+20log10\;\left(\frac{1}{Vadc}\right);$$ (1)

$$b=20log10\sqrt{mean(y^{2}});$$ (2)

a=b−S; (3)

where a = calibrated sound level in dB re 1 μPa; b = uncorrected signal; S = correction factor; h = hydrophone sensitivity (i.e. -186 dBV μPa-1); g = DSG gain (i.e. 20 dB); Vadc = analog-to-digital conversion (i.e. 1 volt); and y = signal.

**Fig 2:**
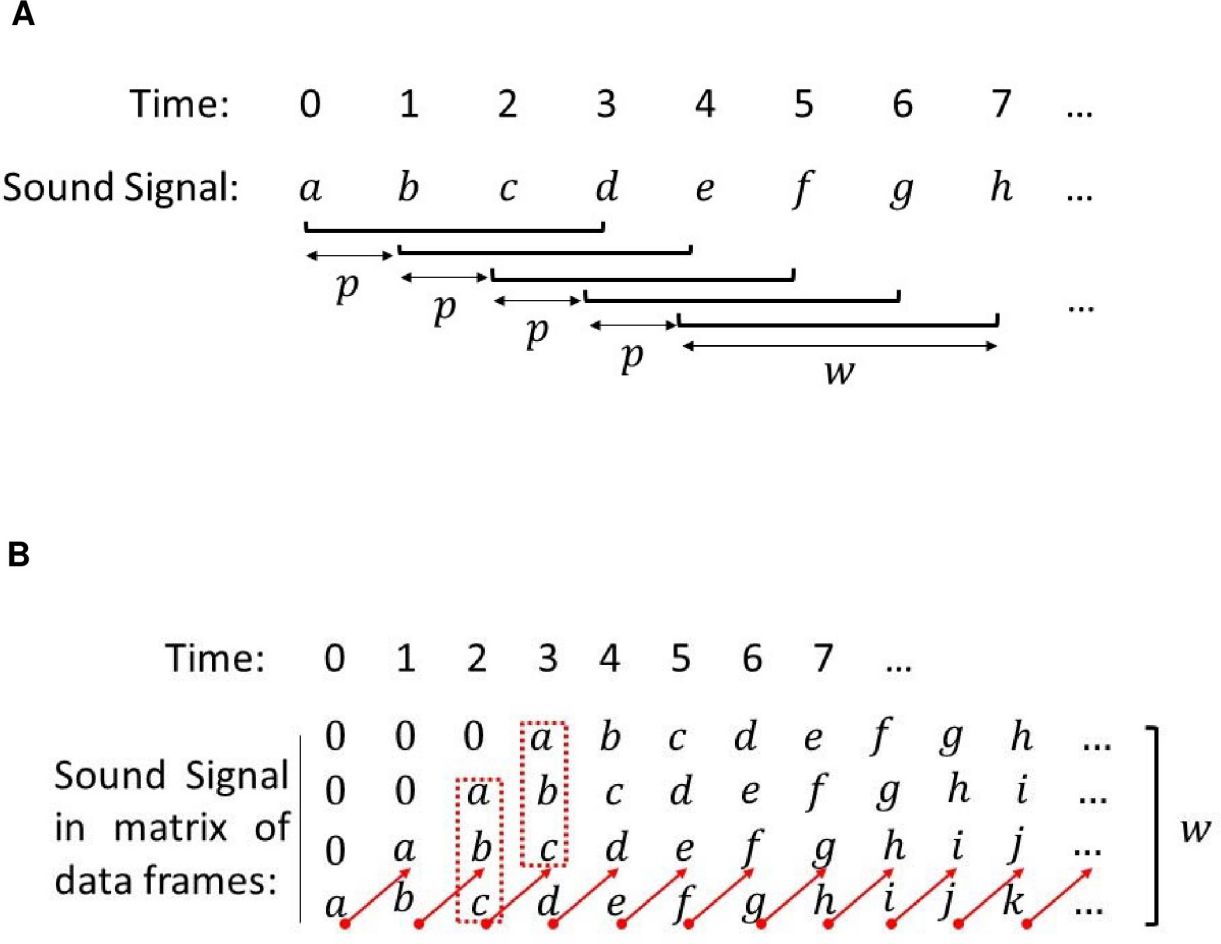
Automatic detection process. (A) Signal data framing method. The array was reorganized into a matrix of data frames using a predefined frame window *w* (e.g. *w* = 4) and parameter *p* (e.g. *p* = 1) to create a repetition of a smaller window of data signals in the data frame. (B) Data framing example. Data signal repeated in the data frame (e.g. *w*—*p* = 3).

For signal detection, our first approach was to reorganize each 2 min wav file into a matrix of data frames using a predefined frame window (i.e. size *w*) (Fig 2A). In addition, we defined a parameter *p* as a repetition of a smaller window of data (Fig 2A). For computational performance purposes, the repetition parameter *p* was adjusted between 1 and the size of the frame window *w*, or p ∈ (1, *w*). Higher values of the repetition parameter (i.e. for example, *p*→*w*) result in fewer repetition data signals and thus a smaller amount of data frames. Consequently, the amount of processing time is reduced. On the other hand, decreased data frames mean lower processing resolution. For a given acoustic signal with sampling frequency *f*_*s*_, the time between neighboring data samples was represented by:

$$\Delta t=t_{n}‑t_{n‑1}=\frac{1}{f_{s}}$$ (4)

where n = 1, 2, 3… When the acoustic signal was restructured with the frame window *w* and repetition parameter *p*, the time between neighboring frames was:

$$\Delta \tau =\tau_{n}‑\tau_{n‑1}=\frac{p}{f_{s}},\;i.e.\;\Delta \tau =p∙\Delta t$$ (5)

When repetition parameter *p* = 1, then Δτ = Δt. According to Eq (5), it is intuitive to use a larger repetition parameter for species with longer calls (e.g. black drum), while for species with short pulses, such as silver perch and red drum, smaller repetition parameters would be recommended in order to capture meaningful acoustic properties.

Similarly to the repetition parameter that impacts the computational processing time and acoustic properties, the frame window size *w* was also critical. The frame window size defines the frequency resolution of the new formed data frame matrix:

$$\Delta f=\frac{f_{s}}{w}$$ (6)

As shown in Eq (6), the larger the frame of the window size, the higher the frequency resolution (or smaller Δf values). For example, for the sampling rate used (i.e. f_s_ = 80 kHz) in this study and a frame window size of *w* = 4096, then the frequency resolution of the resultant data frame matrix would be $\Delta f=\frac{f_{s}}{w}=19.53$ Hz. This approach means that the recorded SPL values of every other 19.53 Hz frequency will be captured in the newly formed data frame matrix (i.e. acoustic properties at frequency ranges between n∙Δf and (n+1)∙Δf, n = 0, 1, 2,… would be neglected). Therefore, the selection of frame window size is critical, especially for those fish species with narrow frequency ranges. For example, black drum calls have a narrow frequency range between 80 and 400 Hz and if a frame window size *w* = 4096 is used, acoustic properties at only 16 frequency instances would be captured and thus could leave out critical property features in the detection process. On the other hand, for silver perch, because of its wider frequency range (i.e. 80 to 5000 Hz), a smaller frame window size would be suitable. With the data frame matrix, signal SPL values were computed for each data frame (i.e. a column of data in the matrix) at a corresponding time instance (Fig 2B). All resultant SPL results for all data frames (i.e. at all the time sequences) were then stitched together to represent a complete spectrum of properties from all sound sources.

A key step in the signal detection process was determining features that represented the calls from each fish species. We randomly selected calls from different stations and seasons (according to our manual verification results) that varied in minimum, maximum, and frequency range, and call duration. This included 51 calls of black drum, 144 calls of silver perch, 145 calls of spotted seatrout (i.e. 36 drums, 91 grunts, and 18 staccato type of calls), and 171 calls of red drum. From these calls, we identified representative calls that differed in these extracted parameters. In this study, we used 6 features for black drum, 29 for silver perch, 9 for spotted seatrout, and 5 for red drum in order to optimize signal detection. These features were saved as a three dimensional (3D) array with rows representing call duration and columns for frequency and each element in the array indicating SPL at the corresponding time and frequency. Essentially, each 3D array represented a 3D feature of the respective call. The detection process involved the comparison of existing features A_m×n_ to all possible instances B_m×n_ within a given 2 min wav file. In order to measure the matching wellness between the feature and detections, we used a parameter s to quantify the detection process for possible successful detections. It was defined as:

$$s=\frac{\sum_{m,n}\left[(A_{m\times n}‑\bar{A}).\times (B_{m\times n}‑\bar{B})\right]}{\sqrt{\sum_{m,n}{\left(A_{m\times n}‑\bar{A}\right)}^{2}\sum_{m,n}{\left(B_{m\times n}‑\bar{B}\right)}^{2}}}$$ (7)

where: $\bar{A}$ and $\bar{B}$ = average of matrix elements; x = operator means element by element matrix multiplication. Based on this approach, we designed a custom script in MATLAB R2017b (MathWorks, Inc., Natick, MA, USA) and “scanned” all the 2 min wav files, searching for calls produced by silver perch, black drum, spotted seatrout, and red drum.

### Statistical analyses

Statistical analyses were performed using SPSS Statistics 24 (IBM Corporation, Armonk, NY, USA) and MATLAB R2017b. To determine if the automatic detector labeled all the acoustic files correctly (i.e. fish-specific calls present or absent in the file), we compared the results from manual and automatic detection by calculating the identification rate (IR) based on the equation [25]:

$$IR=\frac{a-b-c}{a}x\;100\%$$ (8)

where a = total number of files collected; b = total number of files with false negatives; and c = total number of files with false positives. Then, the next step was to compare manual and signal detection quantification by summing calling intensity scores from manual detection and number of calls from automatic detection per night (12:00 to 11:40 the next day) for black drum, silver perch, spotted seatrout, and red drum at stations 4M, 9M, 14M, and 37M. We then performed a Pearson’s correlation test between those sums. For each station and species, we created box and whisker plots of calling intensity score (i.e. 0, 1, 2, or 3) obtained from manually reviewing files versus the number of calls detected via automation. We used a general linear model (GLM) to test which variables (i.e. location, month, day length, lunar phase, temperature anomaly, and tidal range) significantly influenced fish calls from both the manual and automatic detection data sets. Water temperature anomalies were calculated by performing a 30-day moving average on the data and then subtracting it from the observed water temperature data [11]. We used 4 categories to differentiate the lunar cycle: new moon (lunar days 27–4), first quarter (lunar days 5–11), full moon (lunar days 12–19), and third quarter (lunar days 20–26) [31]. Normality of dependent variables was examined by investigating histograms, skewness, and kurtosis of the data. The absolute value of the skewness was < 2 and of the kurtosis was < 7, which indicated that the data were close to a normal distribution [32–34]. If categorical variables significantly influenced sound production, we performed Dunnett’s C tests to determine whether or not group means within each categorical variable were significantly different from each other. In addition, we plotted calling rate for each fish species with corresponding temperature anomaly at each station to determine how temperature fluctuations affected calling rates.

## Results

### Fish call features and automatic detection

**Fig 3:**
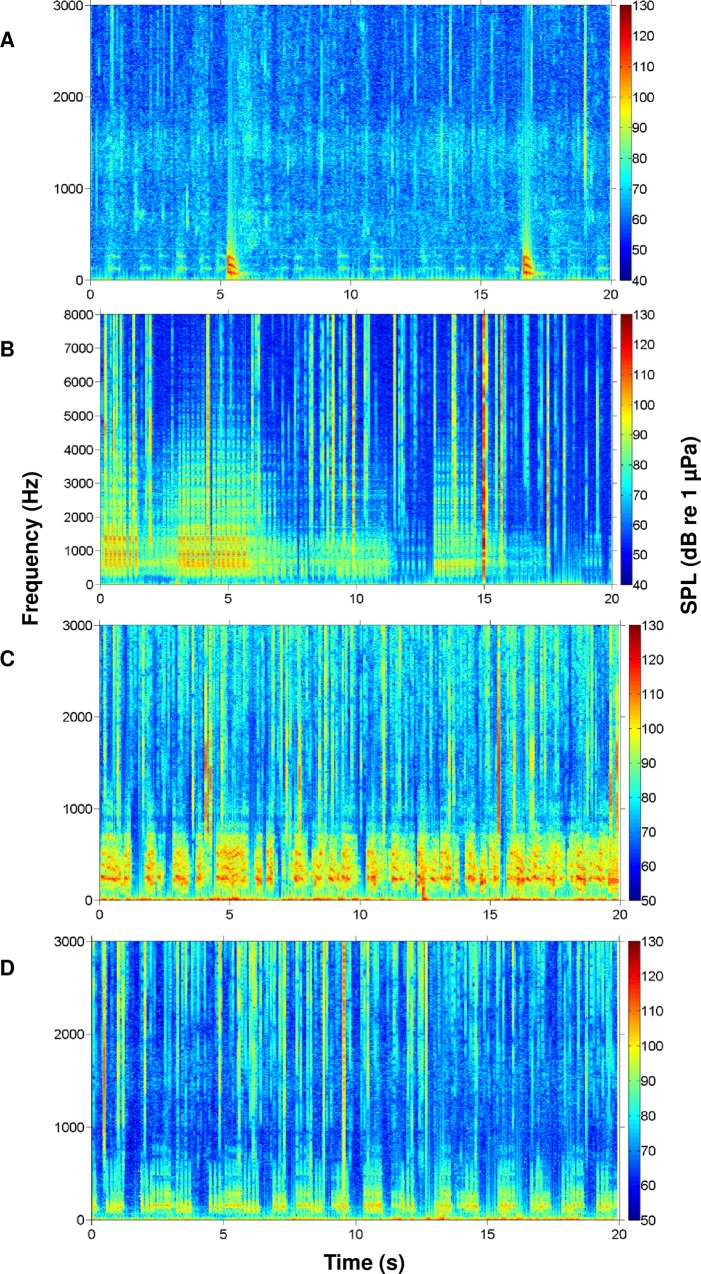
Spectrograms of identified sciaenid fish calls detected in the May River. (A) Black drum *Pogonias cromis*; (B) silver perch *Bairdiella chrysoura*; (C) spotted seatrout *Cynoscion nebulosus*; and (D) red drum *Sciaenops ocellatus*. Spectrograms were created using a rectangular window with window length of 0.1 s and window overlap 90% from original 2 min wav files. Brighter colors indicate higher received sound pressure levels.

**Fig 4:**
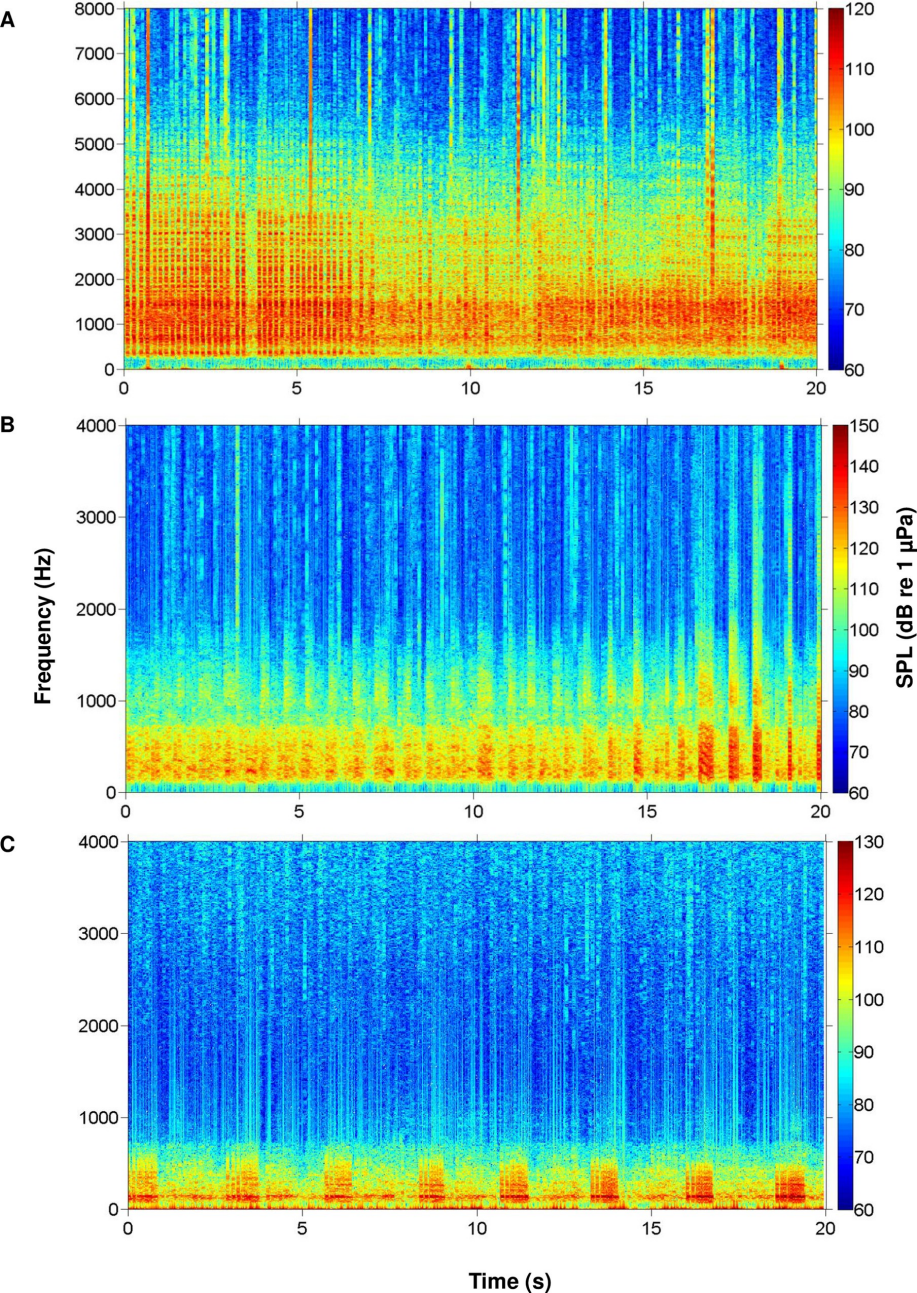
Spectrograms of identified sciaenid fish chorusing detected in the May River. (A) Silver perch *Bairdiella chrysoura*; (B) spotted seatrout *Cynoscion nebulosus*; and (C) red drum *Sciaenops ocellatus*. Spectrograms were created using a rectangular window with window length of 0.1 s and window overlap 90% from original 2 min wav files. Brighter colors indicate higher received sound pressure levels.

**Fig 5:**
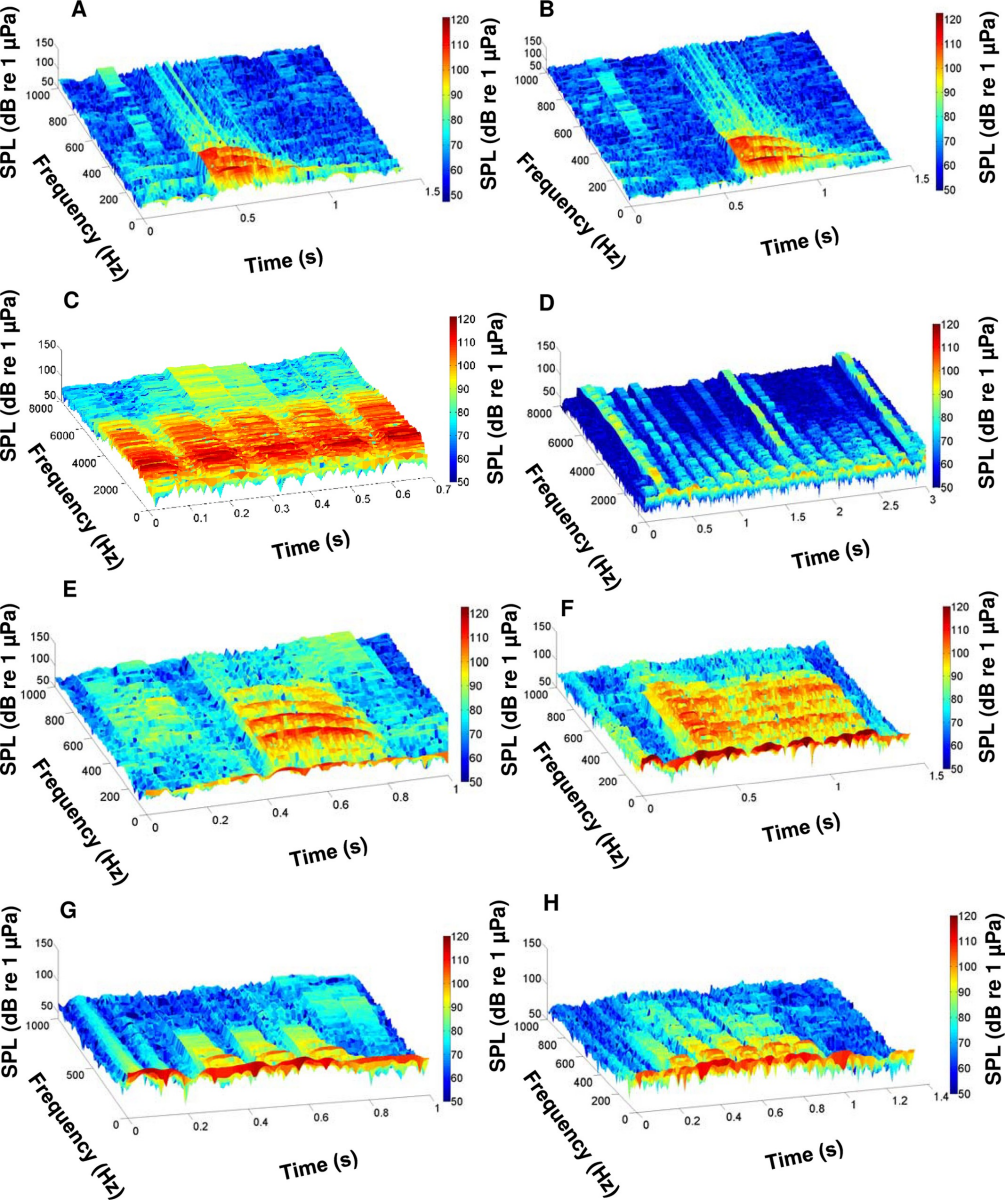
3D spectrogram of representative features for different calls from each sciaenid fish species. (A-B) Two different black drum *Pogonias cromis* calls illustrating harmonic features; (C) silver perch *Bairdiella chrysoura* call with 5 pulses; (D) silver perch call with 13 pulses; (E) spotted seatrout *Cynoscion nebulosus* grunt; (F) spotted seatrout staccato with 21 pulses; (G) red drum *Sciaenops ocellatus* call with 3 pulses: and (H) red drum call with 5 pulses.

**Fig 6:**
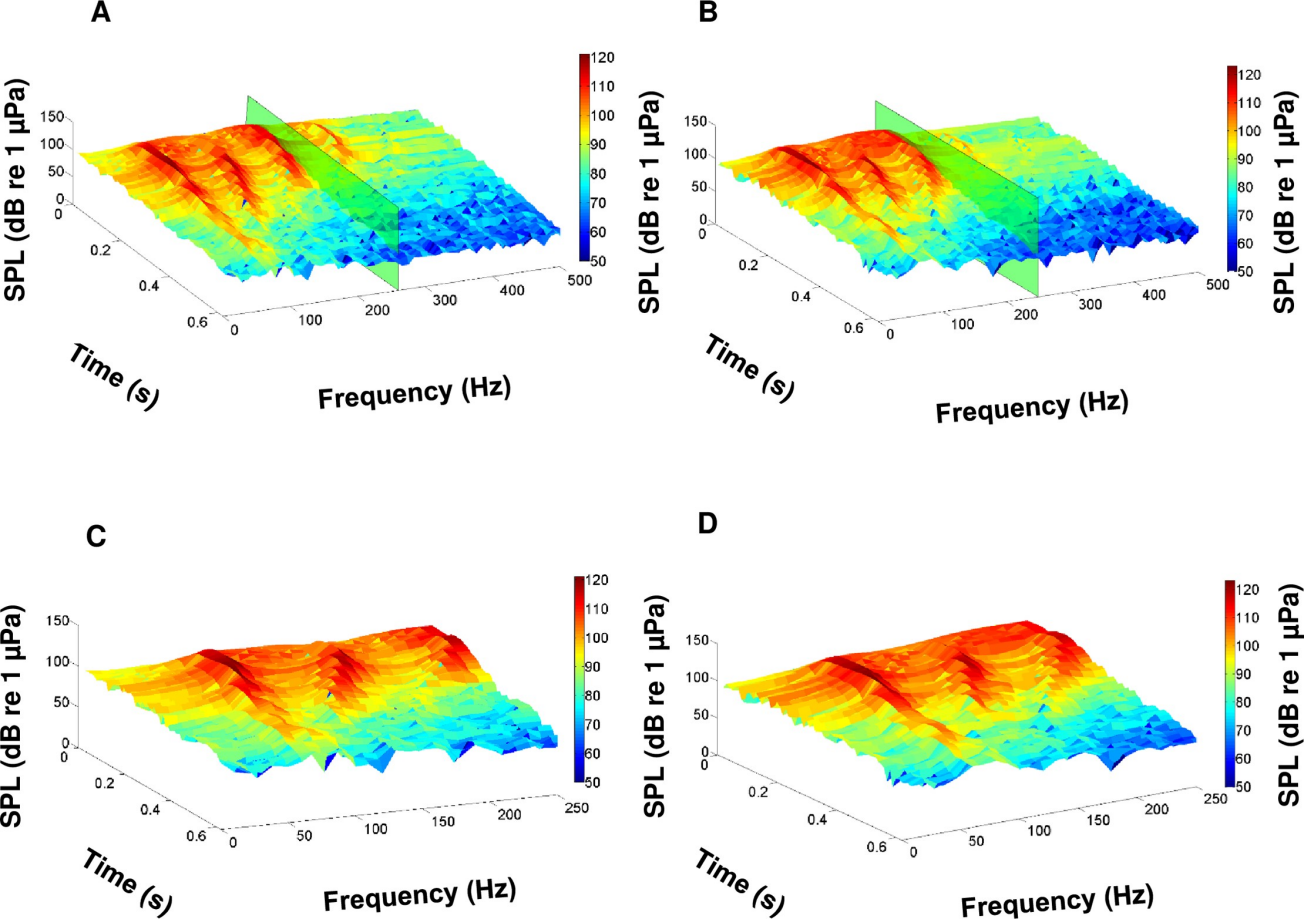
Feature detection process. (A) Feature call of black drum *Pogonias cromis* and (B) call of black drum detected during automatic detection process. Green rectangular boxes show cut off frequency (i.e. above 250 Hz) that was not used as a feature. (C) Feature of black drum call that was used during detection processes and (D) call of black drum that was detected during automatic detection.

**Fig 7:**
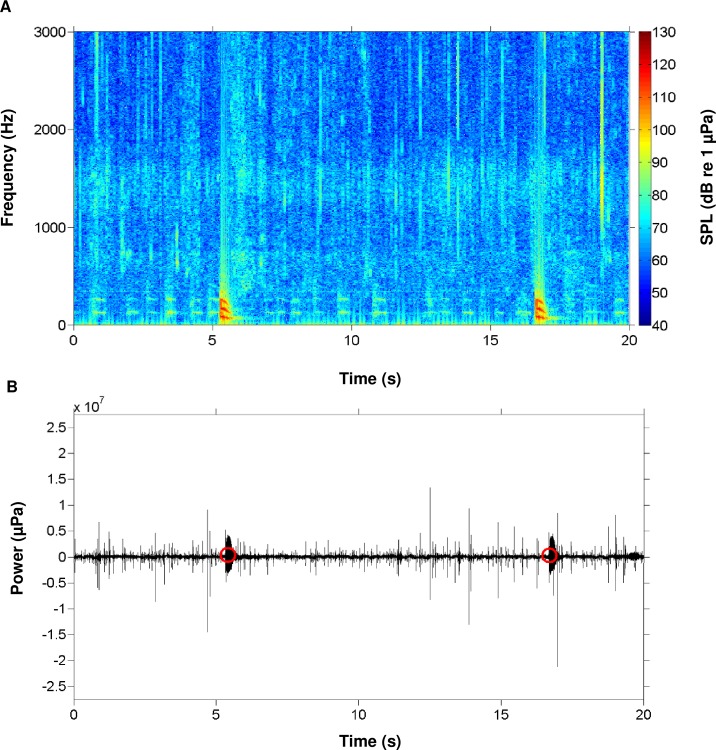
A representative example of call detection using the automatic signal detector. (A) Spectrogram of black drum *Pogonias cromis* calls; and (B) pressure waveform. Red circles indicated calls detected during automatic detection.

During this study, we recorded the calls of multiple fish species native to the May River estuary, SC, USA. We identified the individual calls of oyster toadfish and four sciaenids, including black drum, silver perch, spotted seatrout, red drum, and overlapping calls (i.e. choruses) of silver perch, spotted seatrout, and red drum (Figs 3 and 4). From the recorded calls, we focused on constructing an acoustic library of 3D surface signature features for each sciaenid species (Fig 5). For each fish species, we selected different calls that varied in duration, frequency range (maximum—minimum), peak frequency, and acoustic energy; this diversity in call structure represented different features for that species (Fig 5). Acoustic files were then scanned for these features. For example, a feature representing a black drum call is used to scan an acoustic file (Fig 6A). As a result, a black drum call is found in the acoustic file (Fig 6B). However, the feature has a higher max frequency (i.e. 350 Hz) than the detected call (i.e. 280 Hz) (Fig 6A and 6B, respectively). For this reason, we restrained the feature to detect calls at a lower frequency range by including a partition plane (Fig 6A). Extracting features to a lower frequency allowed us to detect variations in black drum calls (Fig 6C and 6D). These species-specific call features were used to scan all acoustic files collected at four stations along the May River, and the detected occurrences were recorded by the signal detector for each 2 min ‘wav’ file (Fig 7). In total, the observers and the signal detector reviewed 69 696 acoustic files collected at four stations (i.e. 4M, 9M, 14M, and 37M).

### Automatic versus manual detection

**Fig 8:**
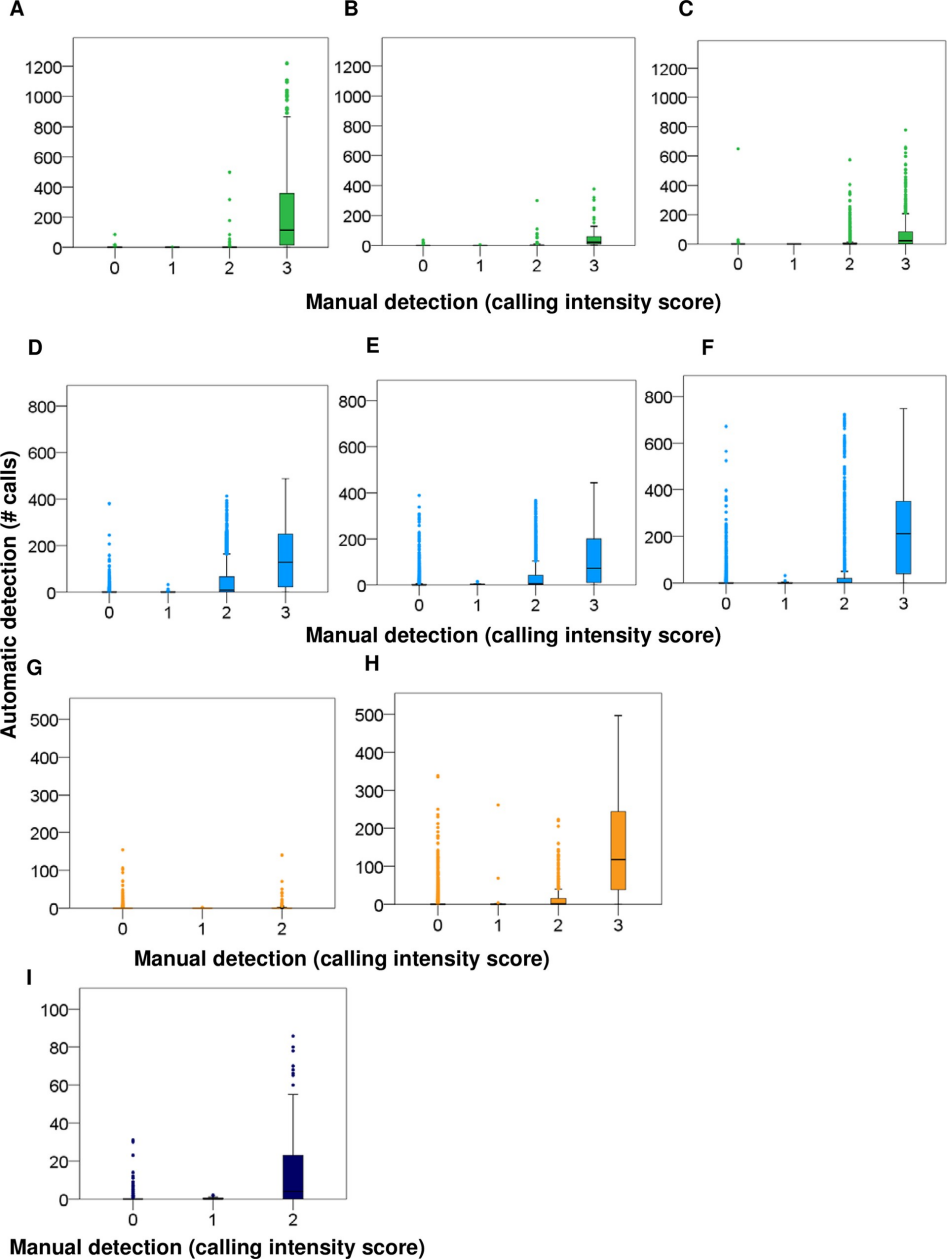
Comparing manual versus automatic detection quantification. Box and whiskers plots, which represent the range of calls quantified by the signal detector for the corresponding intensities (i.e. 0, 1, 2, or 3) provided through manual analysis. Silver perch *Bairdiella chrysoura* at stations (A) 9M, (B) 14M, and (C) 37M; spotted seatrout *Cynoscion nebulosus* at stations (D) 9M, (E) 14M, and (F) 37M; red drum *Sciaenops ocellatus* at stations (G) 9M and (H) 37M; and black drum *Pogonias cromis* at station (I) 37M. The tops and bottoms of each box are the 75^th^ and 25^th^ percentiles of the samples, respectively. The line in the middle of each box is the sample median (50^th^ percentile). Top and bottom whiskers represent the maximum and minimum values, respectively. Values that lie beyond the quartiles by more than 1.5 of the inter-quartile range are marked as outliers.

**Fig 9:**
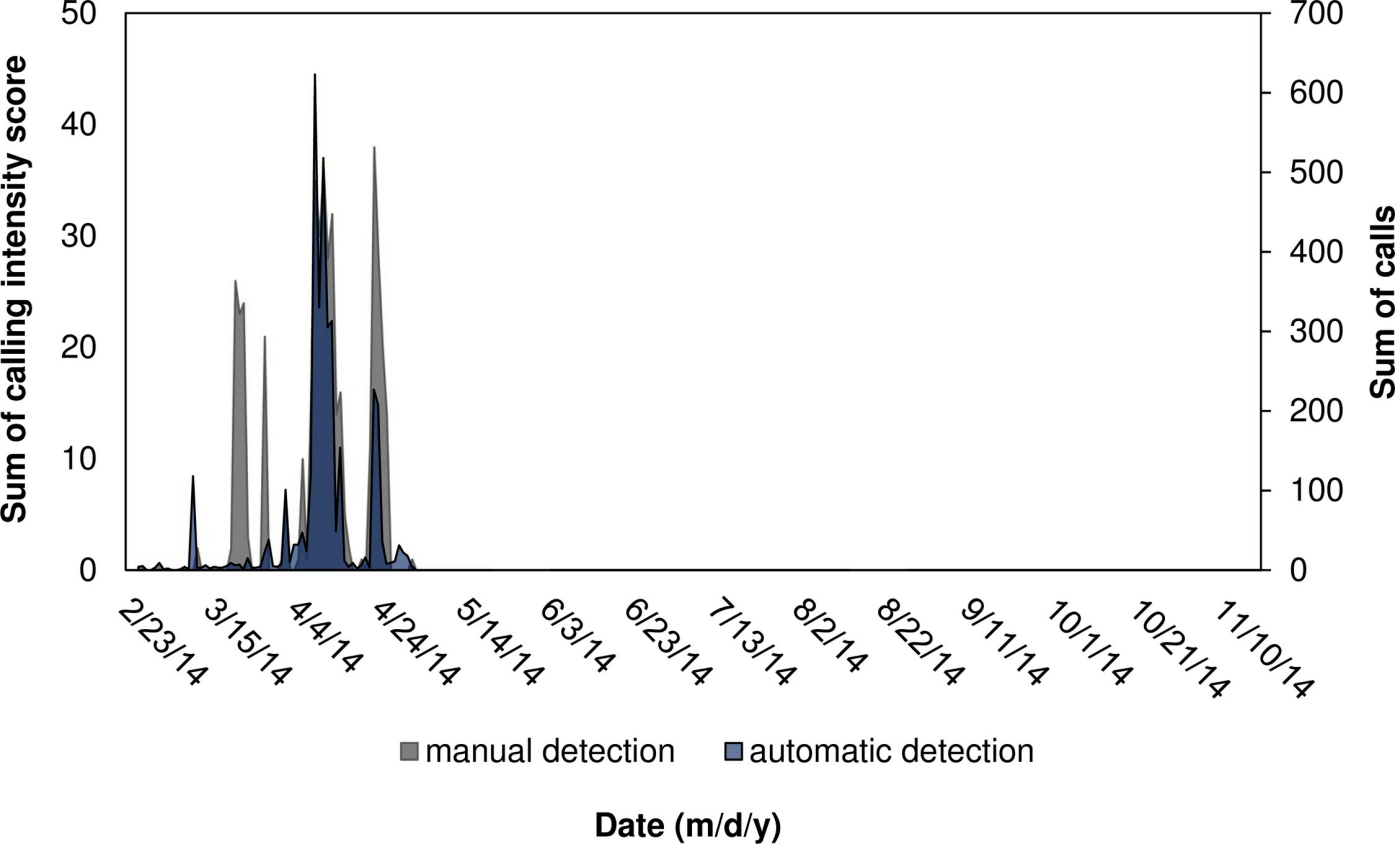
Comparing temporal patterns of black drum calling using manual and automatic detection. Sum of intensity scores obtained from manual detection and sum of calls obtained from automatic detection per evening (i.e. 12:00 to 11:40 of the next day) for black drum *Pogonias cromis* at station 37M.

**Fig 10:**
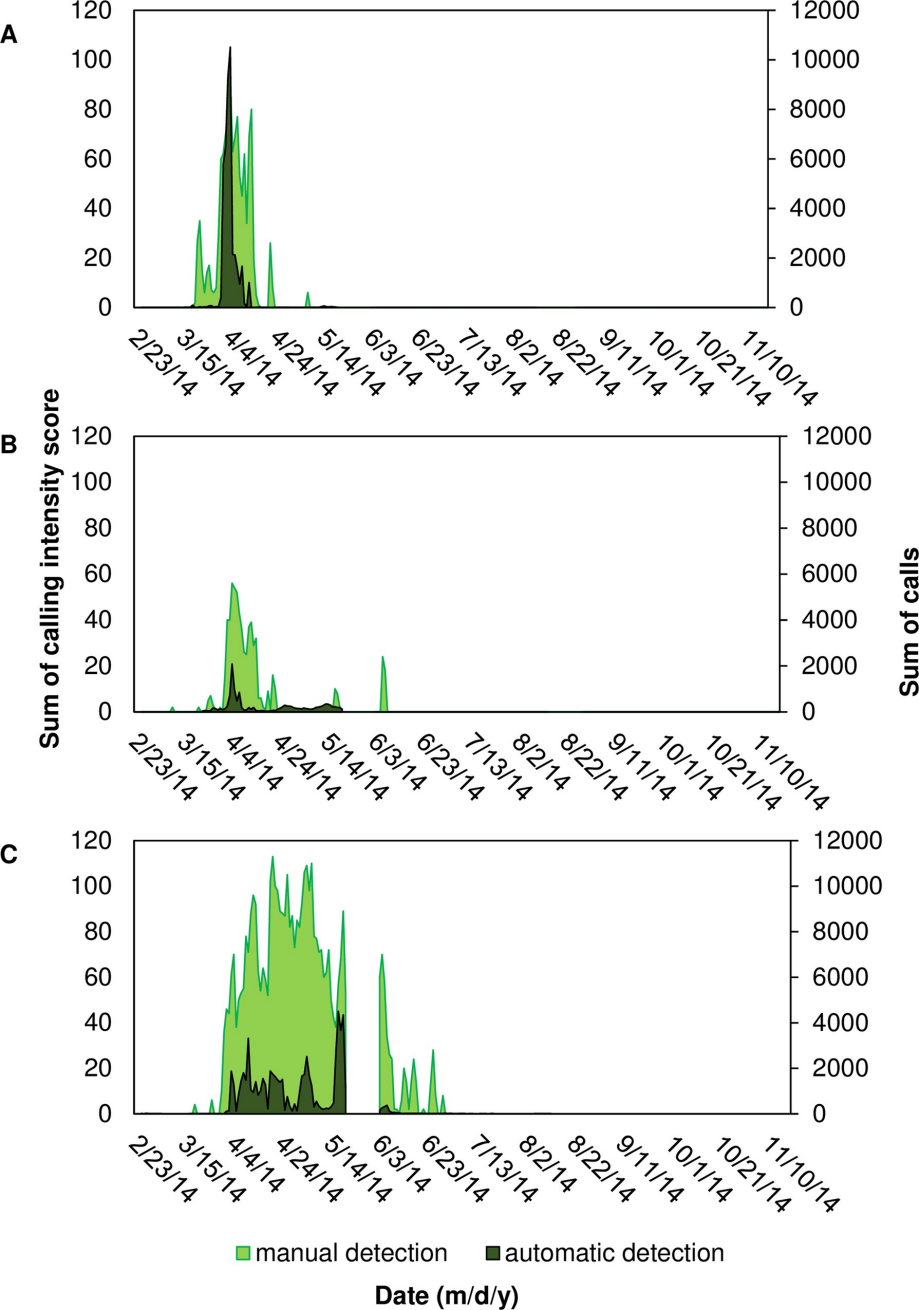
Comparing temporal patterns of silver perch calling using manual and automatic detection. Sum of intensity scores obtained from manual detection and sum of calls obtained from automatic detection per evening (i.e. 12:00 to 11:40 of the next day) for silver perch *Bairdiella chrysoura* at stations (A) 9M; (B) 14M; and (C) 37M. A gap in data (23 May– 4 June 2014) corresponds to a break between deployments due to maintenance of equipment.

**Fig 11:**
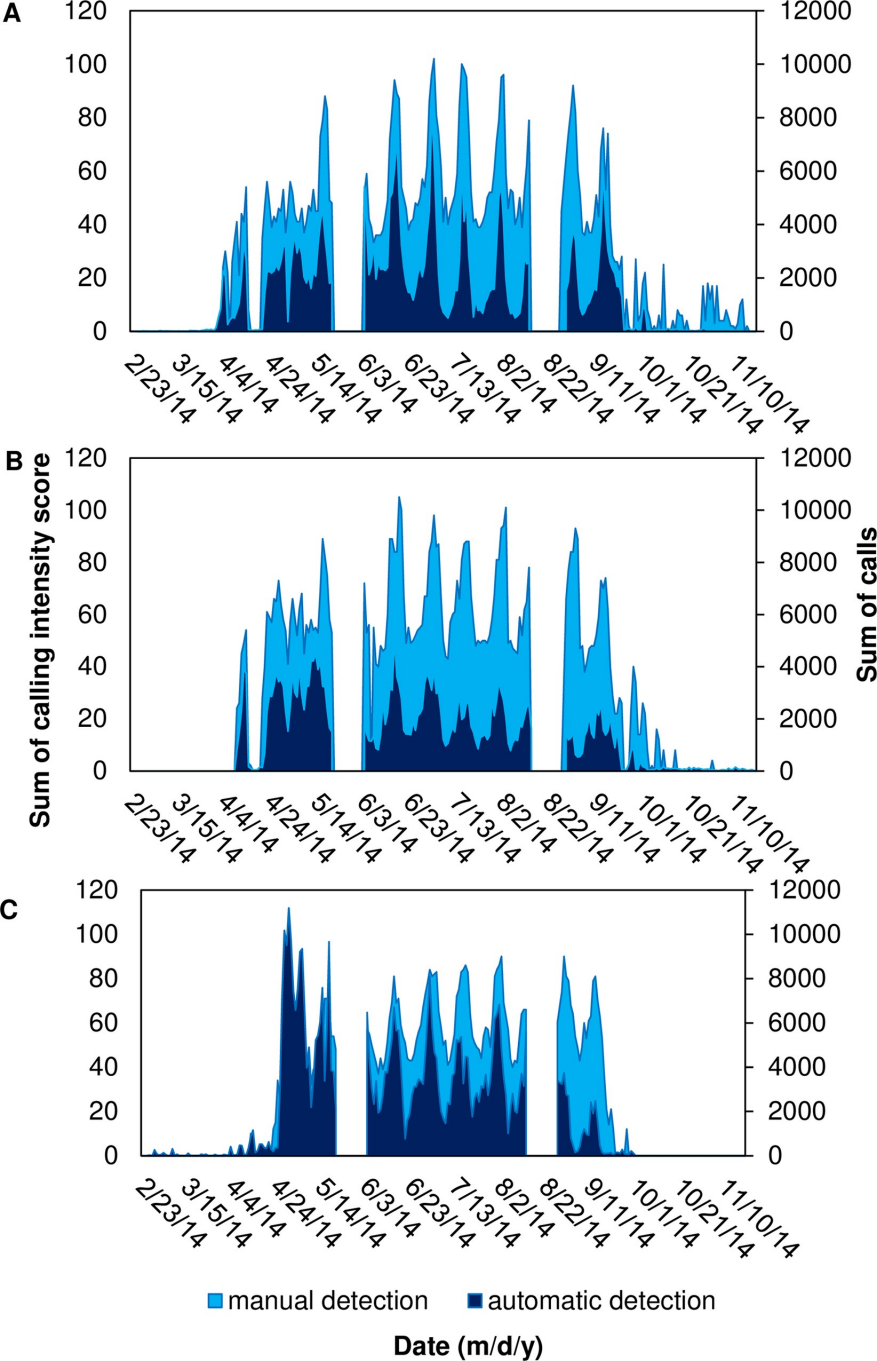
Comparing temporal patterns of spotted seatrout calling using manual and automatic detection. Sum of intensity scores obtained from manual detection and sum of calls obtained from automatic detection per evening (i.e. 12:00 to 11:40 of the next day) for spotted seatrout *Cynoscion nebulosus* at stations (A) 9M; (B) 14M; and (C) 37M. Two gaps in data (23 May– 4 June and 16–29 August 2014) correspond to breaks between deployments due to maintenance of equipment.

**Fig 12:**
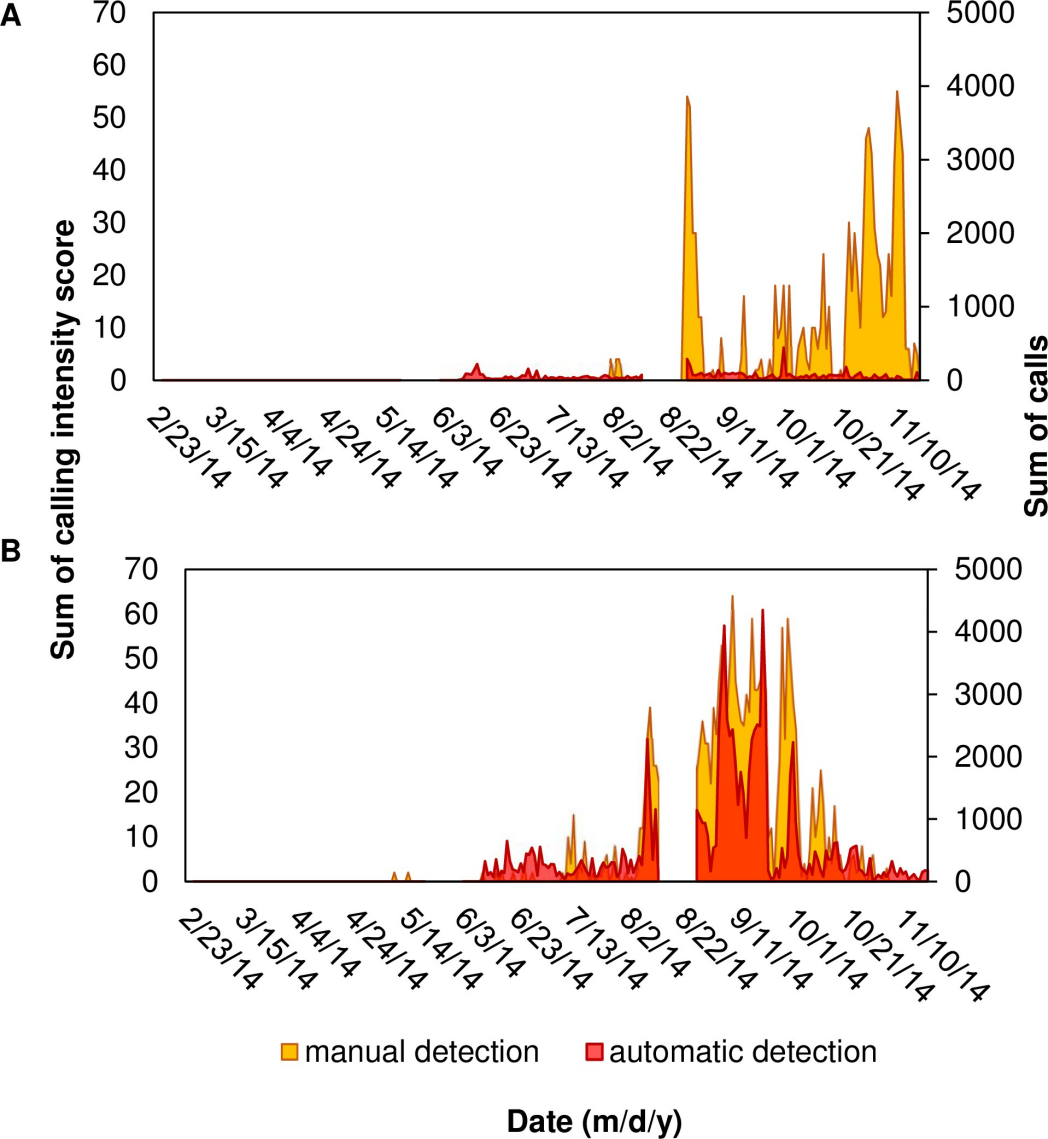
Comparing temporal patterns of red drum calling using manual and automatic detection. Sum of intensity scores obtained from manual detection and sum of calls obtained from automatic detection per evening (i.e. 12:00 to 11:40 of the next day) for red drum *Sciaenops ocellatus* at stations (A) 9M and (B) 37M. A gap in data (16–29 August 2014) corresponds to a break between deployments due to maintenance of equipment.

The signal detector identified a total number of 3 592 calls for black drum; 119 807 for silver perch; 1 101 620 for spotted seatrout; and 10 122 for red drum across all stations (i.e. in all acoustic files recorded). We compared results from manual and automatic detection for each fish species; in most cases, the identification rate was above 80% Table 2. Manual detections were highly correlated with automatic detections for black drum, silver perch, and spotted seatrout at all the stations, and for red drum at station 37M Table 3. The lowest correlation occurred for red drum detections at station 9M Table 3. At this station, the observer marked sporadic calls of red drum but chorusing was never detected. We further investigated how calling rates detected by automatic detection corresponded to the calling categories assigned to each 2 minute wav file by an observer (i.e. manual detection). During manual detection, we used calling categories as a quantitative representation of calling intensity present in each file because choruses contain overlapping calls, which are not possible to manually count. For all fish species, the lowest calling category of 1 had the least number of calls identified by automatic detection, while the highest calling category of 3 had the most number of calls identified by automatic detection (Fig 8). Nightly sums of calling for black drum, silver perch, spotted seatrout, and red drum at stations 9M, 14M, and 37M from manual and automatic detection data sets followed similar spatial and temporal patterns Table 4 (Figs 9–12).

**Table 2:** Automatic identification rate (IR %) at four stations.

	4M	9M	14M	37M
Black drum *Pogonias cromis*	100	100	100	98
Silver perch *Bairdiella chrysoura*	99	96	85	90
Spotted seatrout *Cynoscion nebulosus*	99	61	82	73
Red drum *Sciaenops ocellatus*	100	82	100	81

IR = (#files—#of files with false negative—#of files with false positive / #files) *100%

**Table 3:** Pearson correlation coefficients (rs) and p-value (p) of the sum of the calling intensity scores from manual detection and sum of calls per day from automatic detection.

	Station	rs	p
Black drum *Pogonias cromis*	9M	nd	nd
	14M	nd	nd
	37M	**0.80**	**< 0.001**
			
Silver perch *Bairdiella chrysoura*	9M	**0.70**	**< 0.001**
	14M	**0.68**	**<0.001**
	37M	**0.70**	**< 0.001**
			
Spotted seatrout *Cynoscion nebulosus*	9M	**0.86**	**< 0.001**
	14M	**0.84**	**< 0.001**
	37M	**0.76**	**< 0.001**
			
Red drum *Sciaenops ocellatus*	9M	**0.32**	**< 0.001**
	14M	nd	nd
	37M	**0.82**	**< 0.001**

Values in bold are significant at p < 0.05; nd = calls or chorusing were not detected manually or through automatic detection.

**Table 4:** Results of general linear models that tested the significance of specific factors on fish calling intensity.

	Manual detection			Automatic detection		
Silver perch *Bairdiella chrysoura*	df	F	p	df	F	p
Location	3	64.78	**<0.001**	3	14.59	**<0.001**
Month	4	15.56	**<0.001**	4	17.84	**<0.001**
Day length	1	0.59	0.81	1	1.00	0.32
Lunar phase	3	0.22	0.88	3	0.84	0.47
Tidal range	1	5.68	**0.02**	1	1.20	0.27
Temperature anomaly	1	9.27	**<0.001**	1	6.84	**0.01**
R Squared	0.53			0.42		
Spotted seatrout *Cynoscion nebulosus*						
Location	3	328.87	**<0.001**	3	11.75	**<0.001**
Month	6	56.26	**<0.001**	6	18.94	**<0.001**
Day length	1	154.53	**<0.001**	1	59.40	**<0.001**
Lunar phase	3	11.40	**<0.001**	3	19.64	**<0.001**
Tidal range	1	25.80	**<0.001**	1	30.54	**<0.001**
Temperature anomaly	1	11739.73	**<0.001**	1	24.44	**<0.001**
R Squared	0.73			0.47		

Values in bold are significant at p<0.05.

### Factors influencing calling rates

For both manual and automatic detection, calls of black drum were only detected at station 37M. For silver perch, the mean call intensity scores from manual detection were the highest at 37M (0.24 ± 0.73), then 9M (0.07 ± 0.39), then 14M (0.04 ± 0.29), and the lowest at 4M (0.01 ± 0.13). Automatic detection showed similar spatial patterns, with the highest detections at station 37M (3.65 ± 28.36), then 9M (2.47 ± 40.05), then 14M (0.75 ± 7.22), and the lowest at 4M (i.e. 0 detections). For spotted seatrout, the mean call intensity scores from manual detection were the highest at station 14M (0.47 ± 1.00), followed by 9M (0.44 ± 0.98), 37M (0.41 ± 0.97), and then 4M (0.02 ± 0.17); automatic detection call rates were the highest at station 37M (26.88 ± 93.74), then 9M (19.52 ± 64.09), then 14M (16.82 ± 54.10), and no detections at 4M. Red drum calls were manually detected at stations 9M (0.06 ± 0.34) and 37M (0.12 ± 0.50); we detected red drum chorusing only at station 37M. Red drum call rates quantified by automatic detection were higher at 37M (4.90 ± 29.05) than at station 9M (0.47 ± 3.08).

**Fig 13:**
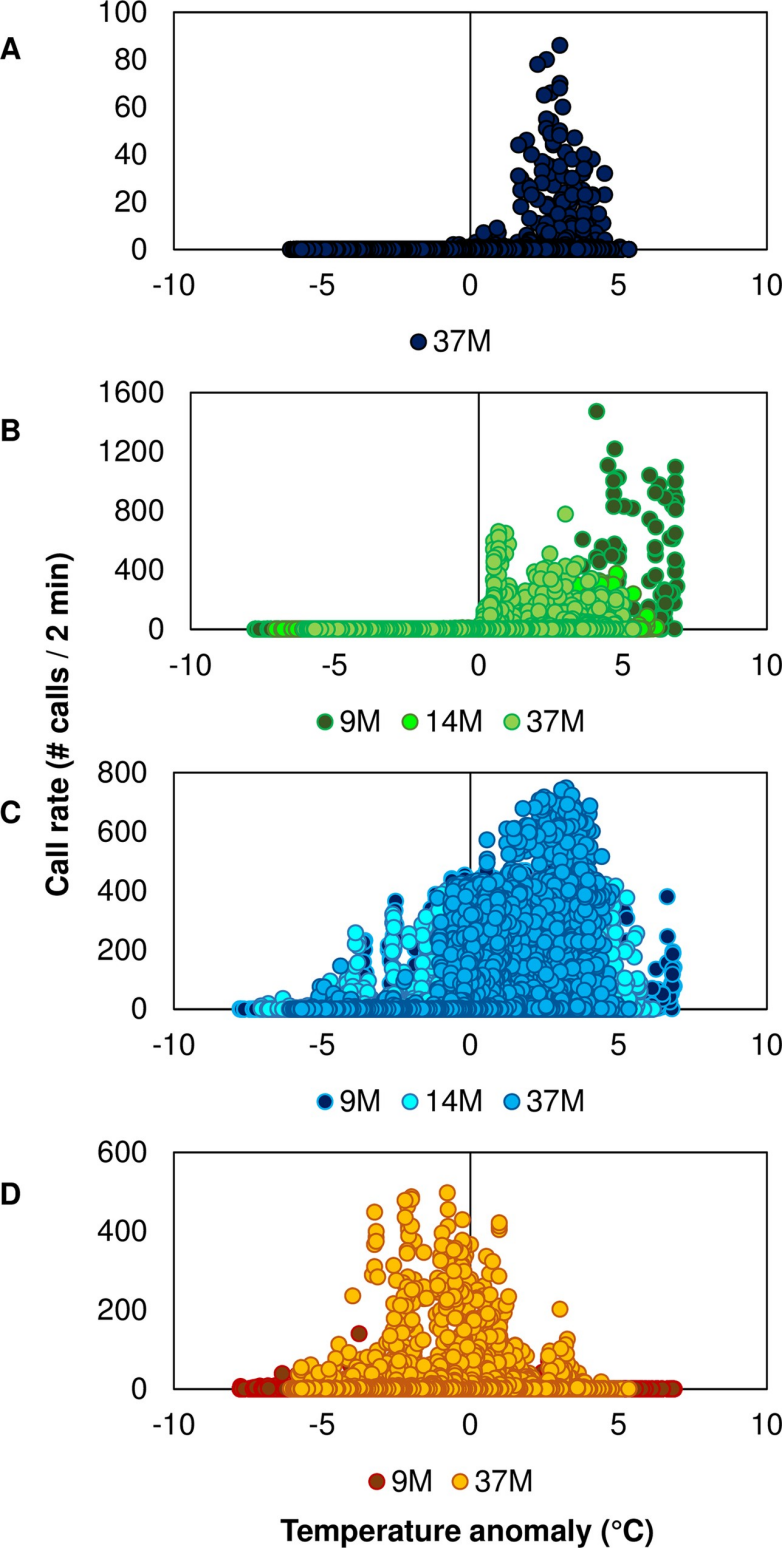
Calling rates determined from automatic detection in relationship to temperature anomaly. (A) Black drum *Pogonias cromis* at station 37M; (B) silver perch *Bairdiella chrysoura* at stations 9M, 14M, and 37M; (C) spotted seatrout *Cynoscion nebulosus* at stations 9M, 14M, and 37M; and (D) red drum *Sciaenops ocellatus* at stations 9M and 37M.

For black drum and silver perch, the highest intensity scores from manual detection and highest calling rates from signal detection (i.e. sums per night) were detected in the spring (March, April, and May); for spotted seatrout, in the summer (May, June, July, and August); and for red drum, in the fall (September and October) Table 4 (Figs 9–12). Temperature anomaly was a significant factor that influenced calling of all fish species (Table 4; Fig 13). Positive temperature anomalies increased calling rates of black drum, silver perch, and spotted seatrout, while negative temperature anomalies increased calling rates of red drum Table 4 (Fig 13). For black drum, calling rates increased when water temperatures reached 13°C in late winter; for silver perch and spotted seatrout, calling rates increased when water temperatures reached 18°C in the spring; for red drum, calling rates increased when water temperatures cooled down to 28°C in the fall. Similar to previous findings [11], day length, lunar phase, and tidal range influenced spotted seatrout calling, and these factors were significant in both manual and automatic detection datasets Table 4.

## Discussion

Passive acoustic monitoring allows scientists to collect massive amounts of data. Naturally, automatic or signal detection methods are becoming increasingly important to improve the efficiency of analysis. Our study sites encompass the complexity and acoustical richness observed in an estuarine system, and we have been acoustically monitoring this estuary since 2013. In the present study, we used signal processing techniques to detect, classify, and quantify calls of four sciaenids (i.e. silver perch, black drum, spotted seatrout, and red drum) that dominate estuarine ecosystems in the Southeast USA. Through manual and automatic analysis, we detected similar spatial patterns with minimal fish calling detected near the source of the May River (i.e. station 4M) and more calling and higher diversity of calls near the mouth (i.e. station 37M). The increase in calling rates, associated with spawning of silver perch in the spring, spotted seatrout in the late spring and summer, and red drum in the fall was consistent with previous studies performed in the May River estuary [8, 11].

### Automatic call detection

We used a diversity of calls produced by black drum, silver perch, spotted seatrout, and red drum to create a species-specific feature library was used to develop a signal detector for automated analysis of large acoustic datasets. The initial step for successful automatic detection was to identify species-specific calls correctly, and the following step was to accurately quantify calls in the 2 min recordings. In marine bioacoustics, most signal detection algorithms have been applied to marine mammal sounds. Techniques have centered on four approaches: (i) feature extraction which involves defining features of a signal that are extracted and then applied for detection and classification purposes; (ii) energy based detection which is very similar to feature extraction but focuses more on energy content; (iii) a third category processes data from extracted features using Hidden Markov Models (HMMs) and Gaussian Mixture Models (GMMs); and (iv) more novel ideas that use information entropy, Eigen-clustering, and neural networks [35–38]. Two dimensional feature based detection has been applied to detect and classify down-sweep calls from sei whales *Balaenoptera borealis* and upcalls from North Atlantic right whales *Eubalaena glacialis* in the Gulf of Maine, MA, USA [39]. During this detection process, the spectrograms were smoothed using a Gaussian smoothing kernel to reduce tonal noise (i.e. ship noise) and the call detection was possible by applying amplitude threshold techniques [39]. Kandia & Stylianou [18] used a Teager-Kaiser energy based system to detect sperm whale *Physeter microcephalus* echolocation clicks. Clicks were detected when the energy of the clicks was higher than the threshold level. Statistical approaches using HMMs and GMMs have been used to detect and classify calls from many marine mammals (e.g. short-beaked common dolphins *Delphinus delphis*, long beaked common dolphins *Delphinus capensis*, Pacific white sided dolphins *Lagenorhynchus obliquidens*, common bottlenose dolphins *Tursiops truncatus*, and killer whales *Orcinus orca*) [20, 40]. Recently, there has been progress in artificial neural networks and machine learning techniques to identify and categorize whistles from captive bottlenose dolphins. These techniques have also been used to identify vocalizations of the North Atlantic right whale in Cape Cod Bay and Great South Channel, MA, USA and clicks originating from Risso’s dolphins *Grampus griseus* in the Gulf of Mexico, USA [37, 41, 23].

Less work has been done in applying automated signal detection to fish calls. Successful signal detectors have been used to detect calls of soniferous fishes including toadfish, red grouper, and striped cusk-eel [24–27, 42]. Recently, Vieira et al. [25] used Hidden Markov Model recognition systems with three features to detect the boatwhistle calls of Louisiana toadfish *Halobatrachus didactylus* in Tagus Estuary, Montijo, Portugal, with an identification rate higher than 90%. However, the detector was not able to correctly identify other calls (i.e. croaks and grunts) produced by this fish species. Ricci et al. [27] used a multi-kernel spectral correlation approach to successfully identify calls of oyster toadfish *Opsanus tau* in Harris Creek Oyster Sanctuary in Chesapeake Bay, MD, USA, by mapping the frequency of the two lowest harmonic signals of the boatwhistle call. Wall et al. [26] studied the spatial distribution of red grouper *Epinephelus morio* calling over the West Florida Shelf, FL, USA and developed an automatic detection algorithm to identify calls and quantify the number of introductory pulses followed by the grunt. Another successful signal detector, based on band-pass filtration, was developed to detect loud repeated pulses (i.e. sound levels over 150 dB re 1 μPa) produced by the striped cusk-eel *Ophidion marginatum* during their reproductive season in Boston Harbor, MA, USA [24, 42].

In our study, the highest identification rate occurred with black drum calls, which are longer in duration and have distinguished harmonics. Other types of calls produced by silver perch, spotted seatrout (drum and staccato), and red drum are shorter and occur in a series of pulses, which might be more challenging for a signal detector to recognize, except for the grunt call of spotted seatrout [25]. For instance, some calls of red drum can consist of three pulses, while others contain five or more and may reflect higher spawning success [14]. This pattern is similar for silver perch, where some calls can be shorter with less pulses, while other calls can be longer with more pulses [43]. Spotted seatrout produce three different types of calls: grunts, drums, and staccatos (i.e. multiple drums where the inter-pulse interval decreases towards the end of the call) [15, 44]. For this study, other challenges of signal detection quantification occur during time periods when silver perch, spotted seatrout, or red drum calling rates are so intense that their pulses overlap creating a chorus. Another challenge occurs during seasons when calling of various species overlaps [8, 11, 45]. For example, in this study, some recorded files contained calls of black drum, silver perch, and spotted seatrout (i.e. during spring deployment), while other files contained calls of spotted seatrout and red drum (i.e. during fall deployment). Acoustic activity of snapping shrimp creates another challenge for signal detection. These snaps, present in every recording, are broadband (i.e. 0–200 kHz) with peak sound pressure levels up to 190 dB re 1 μPa [46]. Snapping shrimp sounds can overlap with fish call pulses and increase the number of false positives or false negatives, depending on the species of fish. However, snapping shrimp snaps have a feature that spans the entire frequency range and the fish feature detector only concentrated on the pulse of their calls. In some cases, boat noise and geophysical noises (e.g. rain and waves) could interfere with automatic detection by masking fish calls and increasing the number of false negatives. Most sciaenids (i.e. black drum, silver perch, and spotted seatrout) increase calling shortly before sunset and continue into the night [e.g. 10, 11, 14, 15]; boat noise is usually not as prevalent during this time frame [47]. However, red drum tend to call earlier in the day and are at a higher risk to boat exposure, which could mask their calls and increase the number of false negatives [11, 47].

### Seasonal changes in calling rates

There is evidence that temperature plays an important factor in the initiation and termination of calling seasons for some fish species [11, 48]. In this study, we showed that increasing water temperatures, in the spring, increased calling rates of black drum, silver perch, and spotted seatrout. Later in the calling season, when the water temperature reached ~23°C and ~28°C, call rates started to decrease for black drum and silver perch, respectively. Black drum calling seasons were recorded for approximately two months, silver perch for about three months, and spotted seatrout called over a period of six months. When the water temperature began to decrease in the fall, the calling rates of red drum increased; their calling season lasted about four months. Ricci et al. [27] showed that the certain parameters of the boatwhistle call produced by oyster toadfish changed during their calling season. The fundamental frequency of their calls increased by ~11 Hz for every 1°C increase in water temperature. It is important to consider changes in call structure (e.g. duration, frequency minimum and maximum, and fundamental frequency) within the calling season and over various temperature regimes, which could easily be incorporated as an output of automatic detection [27, 49, 50].

In this study, we illustrated how passive acoustic monitoring combined with automatic detection is a powerful, noninvasive tool that can be used to quantify the acoustic behavior and spawning potential of a community of soniferous fish species. Signal detection allowed us to quantify the number of calls in each 2 min wav file, which was not possible through manual analysis. Future directions will apply this signal detection process to our long term acoustic data set collected in the May River, SC (i.e. 2013 to present) as well as to the other Southeast USA estuaries that have soundscape monitoring programs. It is possible that this detector may be estuary-specific (i.e. May River, SC, USA) because the calls may differ in acoustic parameters between different study sites, but feature calls representative from different estuaries could be easily added to the acoustic library (i.e. quantitative information and measurements extracted from representative 3D calls). Long-term acoustic monitoring combined with effective automatic detection could potentially be an additional method for monitoring sciaenid spawning and changes in phenology associated with climate variability. These data may be helpful in understanding the productivity and health of estuaries.

## Supporting information

S1 Table The raw data is provided as a Microsoft Excel spreadsheet that contains the following columns of data: Comp folder, file, time, date and time, silver perch (manual detection), spotted seatrout (manual detection), red drum (manual detection), black drum (manual detection), silver perch (automatic detection), spotted seatrout (automatic detection), red drum (automatic detection), black drum (automatic detection). (XLSX)
